# A Glance of p53 Functions in Brain Development, Neural Stem Cells, and Brain Cancer

**DOI:** 10.3390/biology9090285

**Published:** 2020-09-11

**Authors:** Yuqing Xiong, Yun Zhang, Shunbin Xiong, Abie E. Williams-Villalobo

**Affiliations:** 1 Vagelos College of Physicians and Surgeons, Columbia University, New York, NY 10032, USA; yx2516@cumc.columbia.edu; 2 Department of Pharmaceutical Sciences, College of Pharmacy and Health Sciences, Texas Southern University, Houston, TX 77004, USA; a.williams8561@student.tsu.edu; 3 Department of Genetics, University of Texas MD Anderson Cancer Center, Houston, TX 77030, USA; sxiong@mdanderson.org

**Keywords:** p53, brain, development, neural stem cell, glioma, mutation, gain-of-function

## Abstract

p53 is one of the most intensively studied tumor suppressors. It transcriptionally regulates a broad range of genes to modulate a series of cellular events, including DNA damage repair, cell cycle arrest, senescence, apoptosis, ferroptosis, autophagy, and metabolic remodeling, which are fundamental for both development and cancer. This review discusses the role of p53 in brain development, neural stem cell regulation and the mechanisms of inactivating p53 in gliomas. *p53* null or *p53* mutant mice show female biased exencephaly, potentially due to X chromosome inactivation failure and/or hormone-related gene expression. Oxidative cellular status, increased PI3K/Akt signaling, elevated ID1, and metabolism are all implicated in p53-loss induced neurogenesis. However, p53 has also been shown to promote neuronal differentiation. In addition, *p53* mutations are frequently identified in brain tumors, especially glioblastomas. Mechanisms underlying p53 inactivation in brain tumor cells include disruption of p53 protein stability, gene expression and transactivation potential as well as *p53* gene loss or mutation. Loss of p53 function and gain-of-function of mutant p53 are both implicated in brain development and tumor genesis. Further understanding of the role of p53 in the brain may provide therapeutic insights for brain developmental syndromes and cancer.

## 1. Introduction

Over 40 years ago, a ~53-kDa protein was detected with high expression levels in cancer cells [1,2,3], igniting the explosive field of p53 research. Since then, a large number of researches have revealed and are still revealing the potential value and mechanisms of p53 in cancer both in vitro and in vivo [4,5,6]. *p53* gene, which encodes the transcription factor p53, has been identified as the most frequently mutated tumor suppressor gene in human cancers. Mutations in *p53* or activation of pathways that suppress p53 largely contribute to malignant transformation in a variety of cancers. The tumor suppression function of p53 is fundamentally linked to a variety of central cellular events such as DNA damage repair, cell cycle arrest, apoptosis, senescence, autophagy, ferroptosis, and metabolism, mainly through transcriptional regulation of p53 target genes [7,8].

Meanwhile, evidence of p53′s critical roles in development is also mounting [9]. In normal cells, p53 level is maintained low due to ubiquitination and protein degradation mediated by E3-ubiquitin ligase such as MDM2 [10] (For the full name of genes mentioned in this article, please refer to the Supplementary Materials). *Mdm2* null mice exhibited early embryonic lethality, a phenotype that can be readily rescued by deletion of *p53*, indicating the tight regulation of p53 activity during normal embryonic development [11]. In addition, mice bearing a mutant p53 that disrupts Mdm2-p53 interaction and compromises p53 trans-activity, along with the wild-type (WT) *p53* allele, showed late-gestational embryonic lethality associated with a host of developmental phenotypes characteristic of CHARGE (ocular coloboma, heart defects, choanal atresia, retarded growth, and development, genitourinary hypoplasia, and ear abnormalities) phenotypes [12].

**Figure 1:**
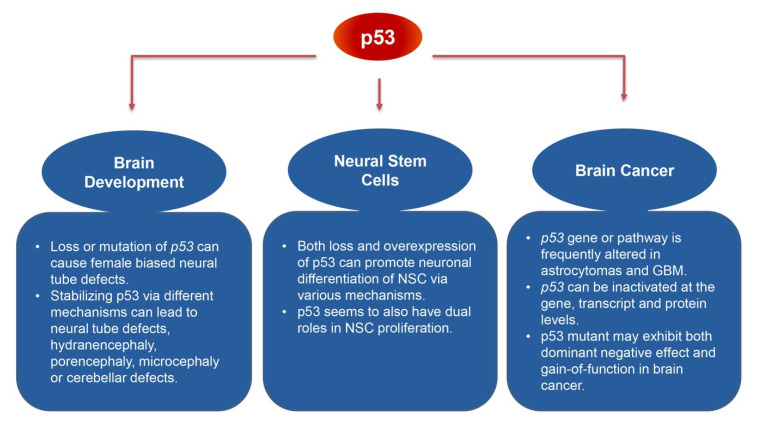
Flow chart outlining the three main topics of this review (p53′s functions in brain development, neural stem cell regulation and brain cancer), and the major specifics discussed underneath each topic.

In particular, p53 has been shown to play pivotal roles in the central nervous system (CNS), during both brain development and brain cancer formation. About 8–23% of *p53* null mice displayed exencephaly, failure of neural tube closure in the region of the mid–brain followed by an outward overgrowth of neural tissue via the eversion of the neural plate [13,14]. Additionally, *p53* mutations are frequently identified in gliomas, which is the most common form of brain tumor [15]. Li-Fraumeni syndrome patients with germline *p53* mutations develop brain tumors [16]. As the importance of p53 in CNS development and cancer has been extensively reviewed previously [15,17,18], this article primarily reviewed studies published during 2005– August of 2020, along with a number of major p53 researches reported more than 15 years ago, which investigated p53′s functions in brain development and neural stem cell regulation, as well as p53 inactivation and its underlying mechanisms in brain tumors (Figure 1). A PubMed search was conducted using keywords including p53 and one of the following terms: brain development, central nervous system development, neural stem cell, brain tumor and brain cancer. In addition, research articles that cited previously reviewed major studies of p53’s roles in the CNS [15,17,18] were screened manually to identify those that are related to the topics of this present review.

## 2. p53 and Brain Development

Neural tube closure is a central issue in developmental biology. The vertebrate neural tube is the precursor of the brain and spinal cord. It forms by progressive adhesion and tissue fusion of paired neural folds at midline along the anteroposterior axis of the embryo, a process requiring tightly coordinated cell proliferation, death and migration [19]. Two early studies have individually shown that a wide variable fraction (8–23%) of female *p53^−^^/^^−^* mice display exencephaly, one common form of neural tube defects (NTDs) [13,14] (Table 1). Other abnormalities seen in *p53^−^^/^^−^* mice include spina bifida (open neural tube in spinal cord), ocular abnormalities and defects in upper incisor tooth formation [13,20,21]. p53-dependent apoptosis seems to only play at most a partial role during normal neural tube closure, as indicated by subtle reduction of apoptosis in the *p53^−^^/^^−^* embryos [14]. A significant number of *p53^−^^/^^−^* mice bypass embryonic lethality [13,14], indicating that p53 is probably only transiently required in specific cells and p53 loss can be compensated by other mechanism(s). In addition, the *p53^N236S/N236S^* mice displayed female-specific exencephaly and spina bifida [22] (Table 1). The occurrence rate of exencephaly was much higher than that in the *p53^−^^/^^−^* females (68.5% vs. 37.1%). Plus, the *p53N236S* mutation led to decreased neuroepithelial differentiation and apoptosis, and increased neuroepithelial proliferation in comparison to *p53* loss. These findings suggest gain-of-function (GOF) of p53N236S in NTDs [22]. Hitherto, to our best knowledge, no comparison of NTDs phenotype has been made between mice containing other *p53* hot spot mutations and *p53* deficient mice. It will be interesting to determine whether the increased NTDs incidence is a p53N236S-specific effect, or it can also be caused by other *p53* hot spot mutations. A variety of mechanisms, such as inhibiting p53 family proteins p63 and p73, have been suggested to underlie the GOF of mutant p53 during tumor genesis and progression [23], and p73-deficient mice show profound defects in brain development, including hydrocephalus and hippocampal dysgenesis [24]. Further investigation is needed to reveal the mechanisms through which p53 loss or GOF regulates neural tube closure. 

It is interesting to notice that the NTDs was primarily found in the female *p53^−^^/^^−^* or *p53^N236S/N236S^* mouse embryos. The cause of this female bias in NTDs has long been postulated to be defective X chromosome inactivation [25], which remained unproven. A recent study, taking advantage of the 100% penetrant, female-exclusive NTDs observed in double loss of pro-apoptotic Bim and p53, shed light on the female-specific functions of p53 during neural tube closure [26]. It showed that in female *p53^−^^/^^−^;Bim^+/^^−^* E9.5 embryonic neural tube samples X chromosome markers Xist and H3K27me3 was decreased and X-linked gene expression was increased at both RNA and protein levels. Moreover, p53 directly bound response elements in the X chromosome inactivation (XCI) center. Interestingly, the incidence of NTDs in the absence of intrinsic apoptosis by completely deleting *Bax*, *Bak*, and/or *Bok* was very low and was not biased to female embryos. These data suggest that loss of p53 that causes stochastic failure in X chromosome inactivation (XCI), instead of lack of developmental apoptosis alone, contributes to the female bias in NTDs. Nevertheless, the molecular mechanism for how XCI failure causes female specific NTDs needs to be further elucidated. The XCI failure has also been suggested to underlie the p53N236S-induced NTDs [22]. Alternatively, p53 has been implicated in regulating estrogen receptors (ER), either by regulating the expression level of ERα [27,28] or competing for the estrogen-responsive target gene promoters [29]. Meanwhile, *p53* is a target gene of ERα [30]. It is noteworthy that sexual dimorphism in the expression of sex hormone receptors in mammalian brains have been observed [31,32]. One can speculate that the development of the female brain, which exhibits gender-specific pattern of sex hormone receptor expression, is probably more heavily dependent on p53 and thus more vulnerable to p53 disruption in comparison to that of the male brain.

Interestingly, while p53 deficiency leads to neural tube abnormality, over-expression of p53 also causes NTDs. Ablation of *Pax-3* resulted in elevated p53 protein level and NTDs in mouse embryos, which could be rescued by loss of p53 [33] (Table 1). Recently, it was shown that zinc deficiency led to increased p53 stabilization and neural tube closure failure, which could be overcome by p53 transcriptional activity inhibitor [34]. 

Additionally, p53 stabilization can also lead to other brain developmental phenotypes. When the negative regulator of p53, MDM2, was ablated specifically in the CNS, mice developed hydranencephaly at embryonic day 12.5 due to apoptosis, whereas the deletion of *MDM4*, another p53 negative regulator, generated a porencephaly phenotype at embryonic day 17.5 because of cell cycle arrest and apoptosis [35] (Table 1). Both phenotypes could be completely rescued by concurrent *p53* deletion. Strikingly, deletion of both *Mdm2* and *Mdm4* genes led to an even earlier and more severe CNS phenotype [35]. These findings indicate the dosage effect of p53 and suggest that p53 level needs to be precisely regulated during brain development. 

In addition, a number of in vivo studies have demonstrated that p53-dependent apoptosis or proliferation defects is profoundly involved in microcephaly caused by removal or mutation of a variety of molecules in mouse CNS (Table 1), including DNA damage repair protein NBN1 [36], the scaffold protein NDE1 [37], centrosomal protein CEP63 [38], tubulin TUBB5 [39], RNA metabolism protein EIF4A3 [40], RNA binding protein RBM8A [40], spliceosome component protein MAGOH [40], citron kinase protein CITK [41], or kinesin-like protein KIF20B [42]. In these mouse models, p53 protein was stabilized and *p53* deletion could largely rescue the microcephaly phenotype [36,37,38,39,40,41,42]. p53 activity also contributes to hypoplastic cerebellum resulted from deletion of *Aspm*, a gene that is mutated in familial microcephaly and in cerebellar granule neuron progenitors [43]. Lastly, the *p53^515C/515C^;Mdm2^−^^/^^−^* mice, which contain stabilized p53R172P, a p53 mutant protein that lacks apoptotic function but is able to elicit a partial cell cycle arrest, displayed proliferative defects in the cerebellum starting from postnatal day 4–6 [44] (Table 1), suggesting that restrained p53-dependent cell cycle arrest is also essential for the normal postnatal cerebellar development. 

It is intriguing that stabilization of WT p53 via deletion or mutation of the above listed different genes can lead to different CNS defects including NTDs, hydranencephaly, porencephaly, and microcephaly (Table 1). Cell-specific and time-dependent p53 stabilization may account for the phenotype variations. In addition, stabilized p53 is probably not the sole determinant of these phenotypes. Alteration of these different genes may not only stabilize p53 but also activate specific partners of p53 that have not been identified by the above studies but are in fact co-essential for causing the specific defects. Since both p53 and the yet-to-be-determined co-factors are required for the abnormal brain developmental phenotypes, suppression of p53 from an aberrantly high level is sufficient for restoring the normal development. This may also explain why the “Super p53” mice that carry an extra, fully functional copy of the *p53* gene did not seem to exhibit any developmental abnormalities [45] and disruption of the Mdm2 E3 ligase function did not generate any brain defects [46], although the dosage effect of p53 may also contribute to the discrepancy. 

In summary, loss/mutation of *p53* or stabilization of p53 may both lead to NTDs. The female specific NTDs may be due to X-chromosome inactivation failure or hormone-related gene regulation. In addition to NTDs, p53 is also involved in other CNS developmental phenotypes such as hydranencephaly, porencephaly, microcephaly, and cerebellar defects. Further investigations are needed to determine why p53 stabilization can cause different brain developmental defects.

## 3. p53 and Neural Stem Cell Regulation

Neural stem cells (NSCs) are multipotent cells, which can self-renew and proliferate without limit, to produce neural progenitor cells (NPCs) that terminally differentiate into neurons, astrocytes and oligodendrocytes [47]. Emerging evidence has indicated that p53 plays important roles in the regulation of NSC/NPC differentiation. In one study, the *p53^−^^/^^−^* mouse brains showed much higher levels of neuron markers Tuj1, MAP2 or NeuN at E11.5, E13.5, or E17.5, but lower levels of astrocyte marker GFAP at E17.5 than WT embryos, suggesting that p53 deficiency promotes neuron differentiation but inhibits astrocyte differentiation at this stage of development [48]. In another study, differentiating E13 p53^−/−^ neural progenitor cells exhibited enhanced expression of neurogenic genes and displayed greater extension of neurites, while astrogliogenesis was not affected [49]. In addition, both GABAergic and glutamatergic differentiation was enhanced in *p53^−/−^* cells. Importantly, in line with the results in cell culture, expression of the neuroblast marker DCX, as well of the GABAergic neuron marker GAD65/GAD67, was elevated between E13 and E16 in the *p53^−/−^* telencephalons, whereas astrogliogenesis was not affected [49]. The suppressive role of p53 in neurogenesis has also been observed in human iPS derived neural embryonic stem (NES) cells, as indicated by an upregulation of neuronal differentiation genes in p53 knockdown (p53KD) NES cells [50]. Furthermore, direct inactivation of p53 by SV40 large T antigen, a short hairpin RNA against *p53*, or genetic ablation of *p53* in *Dgcr8*^−/−^ pluripotent stem cells (PSCs) enabled neural differentiation, while activation of p53 by the MDM2 inhibitor nutlin-3a in WT embryonic stem cells inhibited neural differentiation [51]. 

Efforts have been made to understand the underlying molecular mechanisms through which p53 suppresses neurogenesis. The aforementioned study has suggested that the premature onset of neurogenesis in *p53^−/−^* NPCs is at least in part due to a more oxidative cellular status and increased PI3K/AKT signaling in neural progenitors [49]. In addition, elevated Smad1 expression/activation in the *p53^−/−^* mouse brain and NSC was demonstrated to contribute to the accelerated neuronal differentiation. The expression of ID1 is repressed by p53 in BMP-SMAD1-dependent and independent manners. P53 deficiency caused upregulation of ID1 expression, which in turn accelerated neuronal differentiation of NSCs [48]. Furthermore, downregulation of genes involved in oxidative phosphorylation (OXPHOS) and an upregulation of glycolytic capacity might be also responsible for the increased neuronal differentiation of p53KD NES cells [50], suggesting that p53 acts as a regulator of metabolism in human neural stem cells in addition to its role in cancer metabolism. Considering p53 is a transcription factor that regulates perhaps several thousands of genes with diverse biological functions, it can be speculated that more underlying pathways await to be elucidated. 

Intriguingly, p53 can also promote neuronal differentiation as shown in other studies. For example, overexpression of WT p53 enhanced nerve growth factor-mediated neuronal differentiation in PC12 cells, while mutant p53 or knockdown of endogenous WT p53 inhibited it, the latter of which could be rescued by over expression of WT p53 [52]. SCY1-like 1-binding protein, which decreased p53 protein level, inhibited NGF-mediated neurite outgrowth in PC12 cells [53]. Mechanistically, p53 was suggested to direct interact with and activate the neurotrophin receptor TrkA, stimulating ERK-dependent neuronal differentiation [52,54,55]. 

This puzzling discrepancy in the roles of p53 in neuronal differentiation may be partially due to different model systems (mouse vs. cell culture) being used. In addition, whether p53 promotes NSC/NPC proliferation is also controversial. In several studies, loss of p53 provided a proliferative advantage to neural stem cells/progenitor cells in mouse brain [48,56], which has been suggested to deploy different regulatory networks governing neurogenesis in comparison to human brain [57]. Therefore, caution must be exercised when interpreting the findings. In fact, using human induced pluripotent stem cell-derived neural stem cells, it was demonstrated that instead of promoting cell division, p53 deficiency resulted in slower neuronal stem cell proliferation, potentially due to a prolonged G2 phase [50]. The reduced proliferation rate has also been observed in the p53 knock-down 3D brain organoid system; however, this exploits a different mechanism by accumulating cells in G1 phase at the expense of S phase [50]. The discrepancy between a 2D and 3D system reinforces the notion that brain development is a process under extremely delicate regulation and again warrants the necessity of caution during data interpretation.

In summary, emerging evidence has shown that p53 inhibits neuronal differentiation of NSCs. Oxidative cellular status, increased PI3K/Akt signaling, elevated ID1 and metabolism are all implicated in p53-loss induced neurogenesis. However, p53 has also been shown to promote neuronal differentiation. The role of p53 in NSC/NPC proliferation is also controvertible. Different model systems used in different studies may partially account for these discrepancies. 

## 4. p53 and Brain Cancer 

The most common malignant brain tumors in adults are diffuse gliomas, which are further classified into astrocytomas (WHO Grade II and III), oligodendrogliomas (WHO Grade II and III), oligoastrocytoma (WHO Grade II and III), and glioblastomas (WHO Grade IV) [58]. The common genetic alterations found in astrocytomas occurs in *p53* [59]. In addition, around 12% of Li-Fraumeni syndrome patients with germline *p53* mutations develop brain tumors, including mostly astrocytomas in adults and medulloblastoma in children [16]. Among all brain cancers, glioblastoma multiforme (GBM) is the most aggressive and confers the poorest prognosis. It can be divided into two diseases—primary and secondary GBM. *p53* exhibits different mutation frequency in these two categories. It is mutated in ~30% of primary GBM and identified around in 65% secondary GBM [60]. The number is even higher if alteration of p53 pathways is taken into consideration. For example, 84% of GBM patients and 94.1% of GBM cell lines contain deregulated ARF-MDM2-p53 pathway [60]. Additionally, GBM can be subdivided into different molecular subtypes based on their differing mutational patterns: Proneural, mesenchymal, neural, and classical, which exhibit different prevalence of *p53* mutations (54%, 32%, 21%, and 0%, respectively) [61]. The majority of *p53* mutations is missense mutation. Three hotspot codons, R248, R273 and R175, in the DNA binding domain of p53 represent the highest mutation frequency according to the GBM PanCancer Atlas of The Cancer Genome Atlas (TCGA). In addition to mutations in the coding sequence, splice site mutation of *p53* gene has also been identified in diffuse astrocytomas [62]. Additionally, *p53* promoter methylation was found in 21.4% of 42 primary GB tumors, which, however, was not associated with p53 mRNA or protein level [63]. Instead, *p53* mutation is significantly corelated to p53 protein but not mRNA levels [63]. Thus, the significance of this methylation needs to be further investigated. Nevertheless, the direct evidence for p53′s roles in brain tumorigenesis and progression was obtained from a number of mouse models that carry disrupted *p53* gene alone, or in combination with oncogenes or other inactivated tumor suppressors. These mouse models have been extensively discussed previously [64]. 

A variety of mechanisms of inactivating p53 to promote brain tumor have been revealed (Table 2). Firstly, p53 activity can be dampened by impairing its protein stability [15], including disruption of the ARF/MDM2/MDM4/p53 pathway [65,66,67,68,69,70,71], genomic loss of DNA-damage response factors ATM and CHEK2 [72], and tumor suppressor CHD5 [73]. Secondly, p53 can also be neutralized at the transcriptional level. For example, p53 has been recently identified as a transcriptional target of Parkin. Somatic mutations on *Parkin* have been linked to glioma genesis [74]. Interestingly, p53 was also found to regulate *Parkin* transcription via its DNA binding properties. Parkin expression in mice brain was increased after p53-bearing viral infection [74], suggesting a functional interplay between p53 and Parkin in brain tumorigenesis. IDH1 Arg-132 mutants, R132H/R132Q, which produces extremely high levels of intracellular 2-hydroxyglutarate, suppressed p53 expression through stabilizing hypoxia-inducible factor-2α that activates the expression of *miR-380-5p*, a characterized microRNA that is against *p53* expression [75]. Rescue expression of p53 can inhibit the proliferation rate and impair the resistance of apoptosis induced by doxorubicin in IDH1 R132Q mouse embryonic fibroblast cells [75]. Furthermore, p53 protein levels correlate negatively with IDH1 R132H levels in human glioma samples [75]. This study also suggests that, while p53 mutation can affect cell metabolism, cell metabolism could vice versa impact p53 genetically, resulting in tumor phenotypes [75]. In addition, the transcription factor NF1A, was recently found to have GBM-promoting effects, which were mediated via transcriptional repression of *p53*, *p21*, and *PAI1* through specific NFIA-recognition sequences in their promoters [76]. MicroRNA *141-3p*, the expression of which positively correlates with the malignance degrees of gliomas, has also been suggested to promote glioblastoma progression and temozolomide resistance by altering *p53* expression [77]. Thirdly, overexpression of Bck2L12 protein in primary GBMs has been found to bind to p53 and impair its transactivation potential, inhibiting p53-mediated cellular senescence and cell death [78,79]. Similarly, macrophage migration inhibitory factor (MIF), which was found to be highly expressed in brain tumor-initiating cells (BTIC) in human brain tumor specimen and promote BTIC-induced tumor formation in mouse xenografts, physically interacts with and inhibits p53 [80]. Lastly, it has become evident that impairment of oxidative metabolism via inhibition of complex 1 or decreased mitochondrial DNA copy number could lead to *p53* genetic inactivation and transformation of neural stem cells, which act as the cells of origin for high-grade glioma [81]. 

Mutant p53 has been reported to exhibit GOF in a variety of tumor types such as osteosarcoma, lymphoma, leukemia, and lung and mammary adenocarcinomas [82]. Studies are also revealing that p53 mutant may also possess GOF in brain tumors. In a recent study, p53M237I, which accumulates within amyloid-like p53 oligomers in glioblastoma-derived cells, was indicated to exhibit GOF as cells expressing the M237I-p53 mutant were more resistant to temozolomide (TMZ) treatment than cells expressing WT p53 [83]. In another study, ectopic expression of p53R248L mutation promoted the progression of GBM and enriched inflammation-related signatures [84]. However, one needs to be cautious when elucidating the function of mutant p53 based on comparison between WT p53 tumors and tumors overexpressing mutant p53, as the p53 mutants in these studies may simply exhibit loss of WT tumor suppression function and/or exert a dominant negative effect by suppressing the WT allele. In fact, when p53Y236delta (deletion of p53 codon 236) was transduced into the *p53^+/−^* or *p53^−/−^* primary neuroectodermal cells, which were subsequently transplanted into the brain of the adult WT mice, the presence of Y236delta in transplanted *p53^−/−^* cells had no effect on the tumor frequency and did not affect the tumor latency regardless of the genotype of the transplant. On the other hand, tumor arose from the *p53^+/−^* cells only when transduced with Y236delta. This clearly suggest that Y236delta exerts dominant-negative activity but does not exhibit GOF in the study [85]. In another study, expression of p53Y236delta in the astrocytes of *p53^+/−^* mice significantly reduced the latency of ENU-induced brain tumor but did not alter tumor penetrance [86]. Therefore, GOF mutant p53 is dependent on cell type, cancerous features, and may be also codon-specific.

The current standard treatment for GBM patients is surgery followed by radiotherapy plus concomitant adjuvant chemotherapy with TMZ. However, GBM is still considered incurable [60]. The pivotal role of p53 in GBM makes it a desirable target for therapy. The strategies include prevention of WT p53 degradation, restoration of WT p53 function in mutant p53 tumors and inhibition of GOF mutant p53. A large number of molecules including those that inhibit Mdm2/p53 complex (such as Mdm2 inhibitors AMG232 and RG7112), restore WT p53 structure (such as PRIMA-1 and its analog) and degrade mutant p53 (such as HDAC inhibitors) showed promising outcomes on GBM cells or mouse models and have been discussed previously [60]. However, hitherto none of these molecules have gone to clinic trials for GBM. The upcoming new decade may witness whether manipulation of p53 with some of these molecules will benefit GBM patients. It is also desirable to broaden our understanding of the roles p53 plays in GBM, which may shed light on therapies for this highly malignant brain tumor. 

In summary, *p53* is frequently mutated in astrocytomas and GBM. Mechanisms of inactivating p53 in brain tumors include impairing p53 protein stability, suppressing *p53* gene expression, disrupting p53 transactivation potential and loss or mutation of *p53* gene. GOF of mutant p53 may be involved in brain tumors. However, more efforts are needed to differentiate the GOF and dominate negative effect of mutant p53 in brain tumors. 

## 5. Conclusions

p53 has been one of the most intensively and attractively studied proteins since it was discovered. From a single amino acid alteration, p53 may lose its transcriptional activity and even exert a dominant negative effect by binding to WT p53 protein. Mutant p53 can also experience a GOF and bind to DNA via associating with other transcription factors to alter gene expression. The level of p53 needs to be precisely regulated during development as both loss and overexpression of p53 can lead to abnormal brain phenotypes. Whether p53 promotes or inhibits the neuronal differentiation of NSCs/NPCs remains controversial. *p53* is frequently mutated in brain tumors, with a higher mutation frequency in glioblastomas. The disruption of p53′s functions via impairing its protein stability, gene expression, transactivation potential and gene loss or mutation can lead to brain tumorigenesis. There are still a number of remaining unresolved issues regarding the role of p53 in brain development, NSC and brain cancer. These issues include, but are not limited to, the mechanism(s) of compensating p53 loss during embryonic development, other molecular pathway(s) underlying loss or mutant p53-mediated female-specific NTDs, mechanisms via which p53 promotes or inhibits neuronal differentiation of NSCs, and causes brain cancer. In addition, mechanism(s) that play central roles in the above CNS pathologies, elucidated from studies using in vitro cell cultures and animal models, need to be scrutinized when translated to clinic. More research addressing these issues will provide insight for therapies regarding brain developmental syndromes and cancer mediated by deregulated- or inactivated-p53. 

## Figures and Tables

**Table 1:** Mouse models exhibiting p53-dependent brain developmental phenotypes.

Mouse Models	p53-Dependent Brain Developmental Phenotypes	p53 Status
*p53^−^^/^^−^* [13,14]	Female-specific exencephaly, spina bifida, retinal dysplasia	*p53* deletion
*p53^N236S/N236S^* [22]	Female specific exencephaly and spina bifida	*p53* missense mutation
*p53^−^^/^^−^; Bim^+/^^−^* [26]	100% penetrate, female-exclusive exencephaly	*p53* deletion
*Pax3^Sp/Sp^* [33]	Exencephaly	p53 stabilization
*Mdm2^FM/+^; Nestin-Cre* [35]	Hydranencephaly	p53 stabilization
*Mdm4^FX/+^; Nestin-Cre* [35]	Porencephaly	p53 stabilization
*Nbn^flox/flox^; Nestin-Cre* [36]	Microcephaly	p53 stabilization
*Nde1^−^^/^^−^* [37]	Microcephaly	p53 stabilization
*Cep63^T/T^* [38]	Microcephaly	p53 stabilization
*Tubb5^E401K/E401K^; Nestin-Cre* [39] *Tubb5^flox/+^; Nestin-Cre* [39]	Microcephaly	p53 stabilization
*Eif4a3^flox/+^; Emx1-Cre* [40]	Microcephaly	p53 stabilization
*Rbm8a^flox/+^; Emx1-Cre* [40]	Microcephaly	p53 stabilization
*Magoh^flox/+^; Emx1-Cre* [40]	Microcephaly	p53 stabilization
*CitK^−^^/^^−^* [41]	Microcephaly	p53 stabilization
*Kif20b^m/m^* [42]	Microcephaly	p53 stabilization
*Aspm^SA/SA^* [43] *Aspm^flox/flox^; Math1-Cre* [43]	Microcephaly (hypoplastic cerebellum)	p53 stabilization
*p53^515C/515C^; Mdm2^−^^/^^−^* [44]	Cerebellar defects	Mutant p53 stabilization

Note: p53-dependent brain developmental phenotypes in these models are caused by *p53* deletion, mutation or p53 protein stabilization. Some mouse models may also exhibit non-CNS phenotypes that are not described here.

**Table 2:** Mechanisms of inactivating p53 in brain tumors.

Impairing p53 Protein Stability	Suppressing *p53* Gene Expression	Disrupting p53 Transactivation Potential	Loss or Mutation of *p53* Gene
Gene amplification of MDM2 and MDM4 [66,67] Genetic deletion and methylation of *ARF* [65] Genomic loss of *ATM* [72] Genomic loss of *CHEK2* [72] Genomic loss of *CHD5* [73]	Mutation of *Parkin* [74] IDH1 R132Q mutant (causes activation of the 2-HG/HIF-2a/miR-380-5p pathway) [75] Overexpression of NFIA [76] Overexpression of *miR-141-3p* [77]	Overexpression of Bcl2L12 [78,79] Overexpression of MIF [80]	Inhibition of mitochondrial metabolism [81]

## Supplementary Materials

The following are available online at https://www.mdpi.com/2079-7737/9/9/285/s1, Full gene names for the list of gene symbols used in this article.
