# Supplementary material for: A Glance of p53 Functions in Brain Development, Neural Stem Cells, and Brain Cancer
Source: Biology (Basel). 2020 Sep 11;9(9):285. doi: 10.3390/biology9090285 (PMC7564678; doi:10.3390/biology9090285)
Supplement: Supplementary file 1 [file biology-09-00285-s001.pdf]

Gene list

AKT, Protein kinase B
ARF, Alternative reading frame protein
Aspm, Abnormal spindle-like microcephaly-associated protein
ATM, Ataxia telangiectasia mutated
Bak, Bcl-2 homologous antagonist killer
Bax, Bcl-2-associated X protein
Bck2L12, Bcl-2-like-12
Bim, Bcl-2-like protein 11
BMP, Bone morphogenetic protein
Bok, Bcl-2 related ovarian killer/Bcl-2-like protein 9
Cep63, Centrosomal protein 63
CHD5, Chromodomain helicase DNA binding protein 5
CHEK2, Checkpoint kinase 2
CitK, Citron rho-interacting serine/threonine kinase
DCX, Doublecortin/Doublecortex
Dgcr8, DiGeorge syndrome critical region gene 8
Eif4a3, Eukaryotic translation initiation factor 4A3
Emx1, Empty spiracles homeobox 1
ERK, Extracellular signal-regulated kinase 2
GAD65, Glutamate decarboxylase 2
GAD67, Glutamate decarboxylase 1
GFAP, Glial fibrillary acidic protein
ID1, Inhibitor of differentiation 1
IDH1, Isocitrate dehydrogenase 1
Kif20b, kinesin family member 20B
Magoh, Mago Homolog
MAP2, Microtubule associated protein 2
Math1, Atonal BHLH transcription factor 1
MDM2, Mouse double minute 2
MDM4, Mouse double minute 4
MIF, Macrophage migration inhibitory factor
NBN1, Nijmegen breakage syndrome 1
NDE1, Nuclear distribution protein NudE homolog 1
Nestin, Neuroepithelial stem cell protein
NeuN, RBFOX3/ RNA binding fox-1 homolog 3
NF1A, Nuclear factor 1A
PAI1, Plasminogen activator inhibitor 1
Pax3, Paired box 3
PI3K, Phosphoinositide 3-kinase
Rbm8a, RNA binding motif protein 8A
Smad1, Mothers against decapentaplegic homolog 1
TrkA, Tyrosine kinase receptor A
Tubb, Tubulin beta class I
Tuj1, Neuron-specific class III beta-tubulin
